# Effect of Shielding Gas and Post-Welding Heat Treatment on the Mechanical and Corrosion Performances of Duplex and Super Duplex Stainless Steels’ Low Heat-Input Welded Joints

**DOI:** 10.3390/ma18214818

**Published:** 2025-10-22

**Authors:** Elisa Ferrari, Elena Colombini, Roberto Giovanardi, Francesco Zaniboni, Silvia Gaiani, Paolo Veronesi

**Affiliations:** 1 V System S.r.l., Via XX Settembre 17–19, Fiorano Modenese, 41042 Modena, Italy; elisa.ferrari@unimore.it (E.F.); silvia.gaiani@vsystem.it (S.G.); 2 Department of Engineering “Enzo Ferrari”, Via P. Vivarelli, 10, 41125 Modena, Italy; elena.colombini@unimore.it (E.C.); roberto.giovanardi@unimore.it (R.G.); 291522@studenti.unimore.it (F.Z.)

**Keywords:** duplex, super duplex, GTAW welding, solution treatment, gas shielding

## Abstract

The purpose of this paper is to study the weldability of two specific steels, UNS S31803 (duplex) and UNS S 32760 (super duplex), by making heterogeneous butt joints using gas tungsten arc welding technology. These materials are widely used in applications that take advantage of their superior corrosion resistance, strength, or both, such as chemical plants, oil and gas equipment, offshore sector, marine and other high-chloride environments. Although the joining technique of DSS and SDSS steels is a well-established industrial method, there are several process parameters that can play a key role in the correct execution of welds and in their final achievable properties. Starting from this assumption, this paper investigates some specific aspects such as the influence of heat input and shielding gas composition on the joint’s microstructure and the consequent changes in the ferrite/austenite ratio, also after post-welding heat treatments. Effects on both mechanical and corrosion resistance properties of the alloys are addressed.

## 1. Introduction

Stainless steels can be classified into several different classes, mainly due to the distinction in their microstructure. While each class has a distinct metallurgical phase, stainless steels that belong to the “Duplex” (or DSS) category, show a microstructure obtained from the combination of two distinct phases: ferrite and austenite. The term “duplex” signifies the dual-phase microstructure that contains approximately equal parts of ferrite and austenite. This balanced structure grants duplex stainless steels exceptional mechanical strength and impressive corrosion resistance, particularly against stress corrosion cracking and chloride pitting, which can affect standard austenitic stainless steels [1]. A simple and consolidated method for achieving predictive measurement of stainless steel’s resistance to localized pitting corrosion is based on the Pitting resistance equivalent numbers (PREN) evaluation [2]; this coefficient considers the weight percentage of alloying elements such as chromium, molybdenum and nitrogen, offering a simple way to compare the pitting corrosion resistance of various alloys. While the information is not absolute and should not be used to a great level of precision [3], when combined with data related to costs, availability and processability, PREN can be a useful metric to help to drive the decision-making process and provide a level of confidence. The PREN for duplex stainless steels typically falls between 28 and 38.

Duplex stainless steels may further be divided into three additional subcategories, one of which is a “Super Duplex Stainless Steel” (or SDSS). This family of steels shows a higher alloying element content (typically Cr ≥ 25%) and presents, similar to DSS, a two-phase ferritic–austenitic structure in equal proportions. For SDSS, the PREN values typically range from 40 to 45.

The metallurgy of the DSS and SDSS steel families is complex and requires very close control of composition and heat treatment parameters if mechanical properties and/or corrosion resistance are not to be adversely affected. Particularly, when considering welded structures made from DSS or SDSS steels, it is essential to ensure that the initial microstructure of the base material is not massively altered, and especially that the ratio of austenite/ferrite phases remains as close to 50–50% as possible [4], both in the parent metal and in the weld zone. This precise value is hardly achievable repeatedly, but a range of phase balance is acceptable [5]. Different industrial standards set different limits for acceptable ferrite ranges. For instance, the American Petroleum Institute Technical Report 938-C [6] accepts a ferrite content of 30–65% for the base metal, 40–45% for the HAZ and 25–60% for the welded zone, while the European standard EN ISO 15156-3:2020 [7] allows a ferrite content between 35 and 65% for the base metal and 30–70% for the welded zone.

Moreover, the ferrite content plays a relevant role in the corrosion resistance of such steels, with a lower pitting resistance and higher corrosion rates the higher the ferrite content [8]. For this reason, the achievement of a carefully balanced ferrite content results optimal against stress corrosion cracking in the presence of chlorides and pitting [9].

Whilst phase balance and heat treatment parameters are relatively easy to control during fabrication, this is not the case during welding. The amount of ferrite is dependent not only on composition but also on the cooling rate; fast cooling rates retain more of the ferrite that forms at elevated temperature [10]. Therefore, to minimize the risk of producing very high ferrite levels in the weld metal, it is necessary to ensure that there is a minimum heat input and, therefore, a maximum cooling rate.

Alternatively, post-welding heat treatments (PWHT) can be performed, to recover, at least in part, the desired phase balance.

### Transformations Occurring During Welding and PWHT

SDSS solidify upon slow cooling in a ferritic microstructure, while austenite is precipitated at the solid state below the solidus temperature [11]. Hence, upon welding, a ferritic matrix is formed, hosting austenite as secondary phase. Austenite can precipitate in three different morphologies, depending on the welding conditions [12].

- Allotriomorphic austenite, usually forming at grain boundaries of ferrite δ, requires diffusion of austenite-stabilizing elements towards grain boundaries; it typically occurs in the cooling stage after welding or heat treatment of duplex steels [12];
- Intergranular austenite, which precipitates within the ferrite grains, with nitrides possibly acting as nucleation sites for such austenite type; however, in single pass welding with low heat inputs, it is unlikely to form [13];
- Widmanstätten austenite, forming as needle-like or plate-like structures; it occurs within the austenite phase, and it is favored by slow cooling, and it is commonly observed in the heat-affected zone (HAZ) of welds in duplex stainless steels [14].

Moreover, in the welding region, non equilibrium nitrides can be formed [15] and precipitate inside ferrite grains distant from austenite [16]. Supersaturation of nitrogen can occur upon rapid cooling after welding, triggering the formation of such nitrides. However, in the case of lower cooling rates, other secondary phases like σ or χ are favored, especially in austenite-richer steels [17].

The chemical composition of the fused zone (FZ) is mainly affected by the base material composition, the filler metals, the shielding gases and, partially, by the loss of alloying elements during arc welding. Moreover, nitrogen losses in arc welding of DSS are documented and are ascribed to the de-sorption from the molten pool surface and solidification in the rear part of the melting pool [18]. Recent studies on laser or hybrid MIG/laser welding of DSS demonstrated that steels containing higher percentages of nitrogen are more prone to retain a satisfactory balance between the ferrite and austenite phases after welding [19]. This is also reflected in the selection of the shielding gases, where a mixture of nitrogen containing argon is typically suggested [20]. In a recent study [21], it was found that adding nitrogen to the shielding gas allows a better control over the austenite content, with the volumetric fraction of austenite being proportional to the percentage of nitrogen in the shielding gas, and a suggested percentage of 3.5–4.5% to achieve 50% austenite in the FZ. Nevertheless, the use of commercially pure argon also resulted satisfactory in TIG welding of SDSS, provided the proper welding parameters are used [22].

Concerning the HAZ, an equiaxed microstructure is typically formed at the boundary between the FZ and the base material. Austenite formation on the HAZ, usually occurring at the previous δ ferrite grain boundaries and within the ferrite grains, depends mainly on two factors: chemical composition and cooling rate [23]. Nitrides precipitation in the HAZ can happen, like in the case of the FZ, when the nitrogen content in the ferrite is high, due to rapid cooling [16]. A recent study [24] demonstrated that applying multiple heat cycles and cooling rates varying from 5 to 80 °C/s, the σ phase is the only one precipitating in the ZTA, and its content is affected mainly by the cooling rates, rather than by the number of cycles.

Such precipitation phenomena make annealing post-welding treatments not suggestable for DSS, while solubilization heat treatment followed by rapid cooling can help to decrease the formation of unwanted phases or precipitates. Literature results [25,26] confirmed the beneficial effects of PWHT performed at temperatures in excess of 1000 °C; for instance, PWHT at 1050 °C for 2 h followed by water cooling is able to reduce ferrite formation, with a good austenite–ferrite balance in the FZ [27].

The aim of this study is to investigate the effect of tungsten arc welding parameters like heat input and shielding gas composition and of post-welding solubilization heat treatments on the mechanical and durability properties of a UNS S31803 (duplex) and of a UNS S 32760 (super duplex) stainless steel.

## 2. Materials and Methods

Two sets of duplex stainless steels, namely Duplex UNS S31803 and the super duplex UNS S32760 (Outokumpu, Avesta plant, Avesta, Sweden), whose composition is shown in Table 1, have been used in this study.

**Figure 1:**
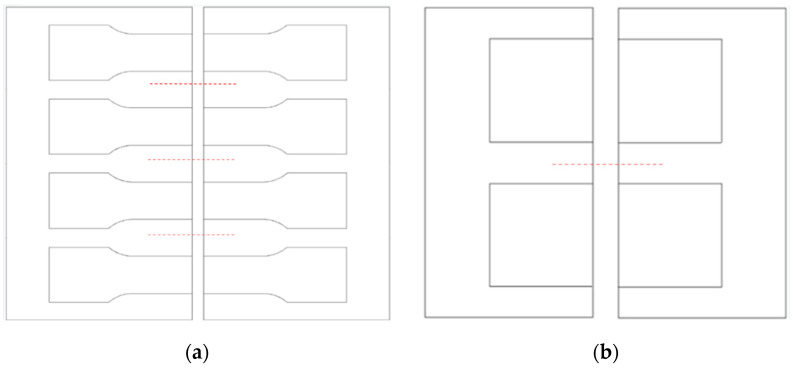
Samples geometry for (**a**) tensile testing, indicating the specimen’s extraction zone; (**b**) corrosion resistance testing, indicating the specimen’s extraction zone. Dotted lines indicate possible stop positions during welding and the position for preliminary spot welding of the plates.

The materials, in the form of cold-rolled sheets with a thickness of 3 mm, solution annealed at 1070 °C and rapidly cooled with water and air by the manufacturer, have been laser cut to the desired shape for welding tests. One set of samples has dimensions of 120 × 65 mm, thus allowing the extraction of four tensile tests specimens, and the second set has dimensions of 210 × 125 mm, allowing the extraction of two specimens for corrosion resistance tests, as shown in Figure 1.

Welding of samples has been performed by manual TIG (Tungsten Inert Gas), using a “Fronius 230I” welding machine (Fronius, Pettenbach, Austria), equipped with a gas lens and a ceramic nozzle, using argon (Air Liquide, Milan, Italy) or argon mixed with 2% nitrogen (H_2_O < 40 ppm, O_2_ < 20 ppm *v*/*v*; Arcal N2-2, Air Liquide, Italy) as shielding gases, with 10 L/min flow. The latter is finalized to help retain the phase composition of the alloys, as described by Tavares et al. [28]. The choice of selecting a 2% nitrogen addition to Ar as shielding gas comes from a recent paper by Akbazadeh et al. [29], focusing on the effects of shielding gas on the wire arc additive manufacturing with duplex stainless steels, where 2% nitrogen addition to Ar provided the highest microhardness, yield strength and tensile strength. As a matter of fact, the level of nitrogen in the shielding gas should match the nitrogen level in the parent metal, and it is different for duplex and super duplex stainless steel. In the case of duplex stainless steel, with a typical nitrogen content of 0.16%, to obtain a similar nitrogen content in the weld metal, the shielding gas should contain from 1.0 to 1.2% of nitrogen. In the case of super duplex stainless steel, with a typical nitrogen content of 0.25%, to obtain a similar nitrogen content in the weld metal, the shielding gas should contain from 2.0 to 2.5% nitrogen [30]. Hence, the choice of a 2% addition is expected to lead to good quality welds in both cases.

Single pass butt welding of the 3 mm thick samples with gap of 0 mm required chamfering of 3 × 45° and the use of a specially designed welding mask with clamps. The welding mask is made of steel plates, with a bottom plate having dimensions of 270 × 230 × 10 mm, on top of which are mounted two smaller plates (230 × 130 × 10 mm) positioned 10 mm apart one from the other, in order to provide a channel for the protection gas (2 L/min) running below the samples to be welded.

Two different filler materials have been used, namely, ER 2209 for duplex S31803 and ER2594 for super duplex S32760 (Commersald, Modena, Italy), whose composition is shown in Table 2.

Welding edges have been carefully cleaned with acetone prior to welding, which has been conducted manually with an average speed of 1.5 or 2 mm/s depending on the shielding gas used, in order to achieve an arc linear energy density of approximately 0.5 kJ/mm for all the tested conditions. Such arc linear energy density has been selected in agreement with the literature results [13], which suggest minimum values for low-thickness welds (like the ones of the present study) of 0.5 kJ/mm, and up to 3 kJ/mm for thicker samples. In this study, we decided to operate at minimum linear energy density because too-high energy densities could lead to the unwanted formation of intermetallic phases [29]. Moreover, the ISO 15156 standard does not allow for more than 1% of deleterious phases in the weld region for such steels.

Table 3 shows the TIG welding parameters used and the calculated arc linear energy density, given by the product of current and voltage divided by the welding speed and a conversion factor 1000 [31].

Tensile test and corrosion test specimens have been laser cut from the welded plates using a Senfeg Laser SF3015HM cutting machine (Senfeg, Jinan, China) with 7 m/min speed, head height of 0.5 mm and nitrogen shielding with 10 bar pressure.

A half of the samples has been subjected to post-welding heat treatment in a vacuum furnace, consisting of heating at 1120 °C at an average speed of 6 °C/min, followed by holding for 1 h at such temperature and cooling to room temperature under argon flux at 6 Atm pressure with an average 0.8 °C/s cooling rate, maximum cooling rate of 150 °C/min in the 1120–500 °C temperature range. This cooling rate was selected considering that the precipitation kinetics of secondary phases in the critical temperature range strongly determines the minimum requested cooling rate. Literature results suggest that for duplex stainless steels with low chromium content, the critical cooling rate is about 0.3 °C/s, while for higher chromium and molybdenum contents it is about 0.1 °C/s [32,33].

The heat treatments have been conducted on a TAV vacuum unit H6 S (chamber dimensions of 600 *×* 600 *×* 900 mm and maximum Ar pressure during cooling of 11.5 bar). Heat treatment has been performed using a charge thermocouple, i.e., a thermocouple positioned in contact with one of the samples.

Metallographic observation of the samples has been performed on resin-mounted samples, polished and lapped, using a Nikon SMZ 745T stereographic microscope (Nikisol Europe, Amstelveen, The Netherlands) and a Nikon ECLIPSE LV150NL metallographic microscope (Nikon Europe, The Netherlands). Specimens were prepared through sequential grinding and polishing. Planar grinding was performed using a coarse abrasive disk under water lubrication (2–3 min, ~20 N load). Intermediate polishing employed a disk lubricated with water drops and a 9 µm diamond suspension. Final polishing was conducted on a soft cloth disk with colloidal silica suspension and water flow to obtain a mirror-like surface.

Subsequently, samples were cleaned in an ultrasonic bath. The specimens, immersed in ethanol within a glass beaker, were sonicated in distilled water for 5 min to remove polishing residues. Image analysis has been performed using the ImageJ 1.54n software. A fixed illumination of samples under the microscope allowed us to achieve micrographs with similar brightness, and a threshold filter was applied on all the acquired images, using the same greyscale value. Image analysis was performed on the whole micrographs, including possible features on the edge of the micrographs. Microhardness tests have been performed on polished samples using a Wilson VH1202 Vickers hardness testing machine (Buheler, Plymouth, MN, USA) applying 500 g load for 12 s. Multiple indentations (15) have been performed, spaced by 1.3 mm, resulting in 5 indentations in the weld zone, 2 indentations in the heat-affected zone (HAZ) and the remaining in the base metal. The applied load of 500 g has been selected according to literature results [32,33]. Tensile tests have been performed according to ISO 6892-1: 2019 [34] using an INSTRON 5982L9379 Universal testing system machine (Instron, Norwood, MA, USA) on standard specimens with a useful length of 80 mm.

Corrosion resistance of the samples has been evaluated by potentiodynamic anodic polarization tests according to ASTM-G5 [35]. Laser cut samples have been polished with acetone and immersed in a saline solution (3.5% NaCl mass/vol), chosen to reproduce one of the typical applications of such steels in marine environments. The corrosion cell (flat cell) has a 1.0 cm^2^ diameter opening on one side, used to position the sample (Working Electrode, WE) and expose it to the saline solution in presence of a Reference Electrode (Saturated Silver Chloride Electrode, SSCE) and a Counter Electrode, made of a platinum mesh wire with a much larger surface area with respect to the WE, allowing currents to flow in the cell without limiting the reactions taking place at the WE. Measurements have been acquired using a VersaSTAT3 Potentiostat Galvanostat (Ametek, Berwyn, PA, USA), according to the following procedure:

- Open Circuit Potential (OCP) acquisition, i.e., measurement of the voltage in open circuit conditions to determine the equilibrium potential of the sample; this measurement was performed for 300 s, considering the final (stable) potential reached by the sample as the equilibrium potential (OCP);
- Potentiodynamic polarization test, measuring current as a function of the WE potential, varying from −0.4 V (vs. OCP) to 1.6 V (vs. OCP), with a scan rate of 0.0004 V/s, according to ASTM G5 standard.

This procedure allows to determine the equilibrium potential, the pitting potential and the passivation zone, with its average passivation current.

Measurements have been taken on the base metals and, for each sample, on the melted zone (FZ) and on the HAZ.

Due to the wide range of conditions tested, the following sample identification will be used in the text: Material_Shielding gas_Post welding heat treatment region, with the following:

- “Material” with values D or SD for duplex or super duplex;
- “Shielding gas” with values A for argon or ArN2 for argon + 2% nitrogen;
- “Post welding heat treatment” assuming treated (t) or not treated (nt) values;
- “Region” with values BASE, HAZ, FZ depending on the tested zone.

## 3. Results and Discussion

All samples, four per each condition, have been tested in terms of microstructure, mechanical properties and corrosion resistance.

### 3.1. Microstructure

**Figure 2:**
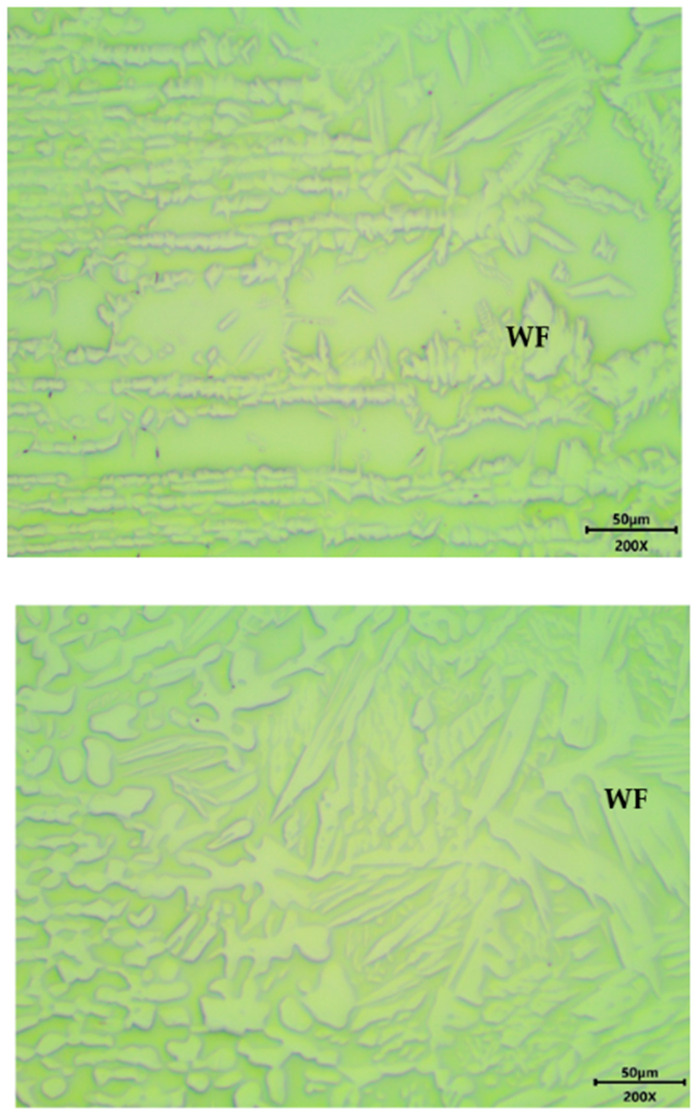
Microstructure in the HAZ in the case of D_Ar samples, without (**top**) and with (**bottom**) heat treatment. Base metal is on the left side of each picture, while the HAZ is on the right side.

Optical microscope micrographs of the polished and etched duplex samples, welded using argon, treated and not heat-treated, in the HAZ, are shown in Figure 2.

Figure 2 clearly indicates that the original banded microstructure, with alternating layers of ferrite and austenite, undergoes severe modification in the HAZ, with extensive grain growth, possible modification of the phase balance and formation of primary ferrite grains at former austenite grain boundaries and growing in a Widmanstätten pattern (WF). Moreover, the heat treatment effects are clearly visible in Figure 2 (bottom), where the base metal also underwent recrystallization and grain growth.

**Figure 3:**
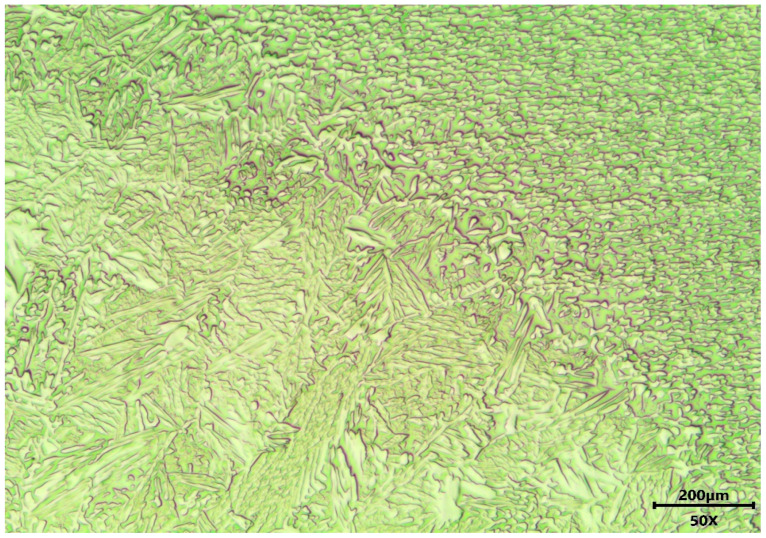
Microstructure in the case of SD_Ar_t samples. Base metal is on the right side, while the FZ is on the left side.

Similar results have been achieved in the case of SD_Ar samples, as shown at lower magnification in Figure 3, depicting an SD_Ar_t sample.

**Figure 4:**
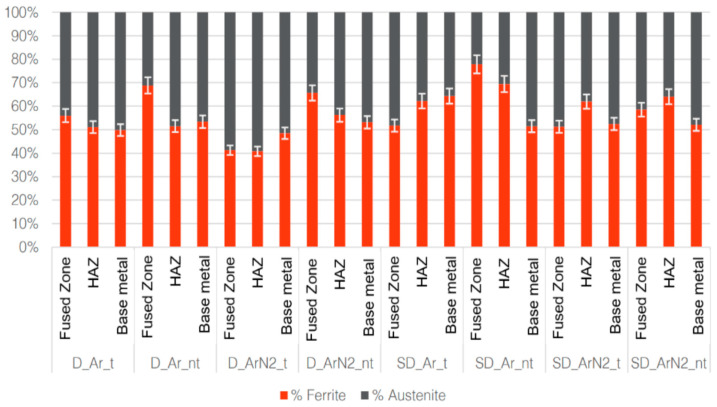
Ferrite and austenite volumetric content in FZ, HAZ and base metal as a function of type of steel (D, SD), gas used (Ar or Ar2%N_2_) and post-welding heat treatments (t).

Noticeably, Figure 3 shows another important aspect of the welds investigated, i.e., the moderate extension of the HAZ. This is typical of welds with moderate to low heat input, as demonstrated also by other authors using a similar energy density [34]. Moreover, in all cases, the ferrite content in the FZ appears to have increased with respect to the base metal, as confirmed by Figure 4, obtained by image analysis on 10 different micrographs per sample.

In all samples not subjected to heat treatment, in both the HAZ and more clearly in the FZ, the ferrite content is increased with respect to the average 50% of the base metal. The effects of the heat treatment are particularly evident in the FZ, lowering its ferrite content. This is expected to be reflected in the corrosion resistance tests, discussed later in the paper.

Concerning the gas mixture used, while the addition of nitrogen has moderate effects on the phase balance in duplex stainless steels (D) with respect to Argon gas alone, in the case of super duplex ones (SD), a lower percentage of ferrite is encountered in both the FZ and HAZ, with the FZ having almost the same ferrite content of the base metal. This suggests that the use of nitrogen in TIG welding can be beneficial to retain the desired phase balance in such steels.

Moreover, on average, the ferrite content in the FZ and HAZ of welded duplex samples (53.90%) and super duplex ones (62.13%) is different, and this can also affect mechanical properties, as discussed later.

### 3.2. Microhardness

Microhardness has been measured along straight lines starting and ending in the base metal and passing through the HAZ and FZ. Examples of the microhardness profiles for each set of samples are provided as Supplementary Materials.

**Figure 5:**
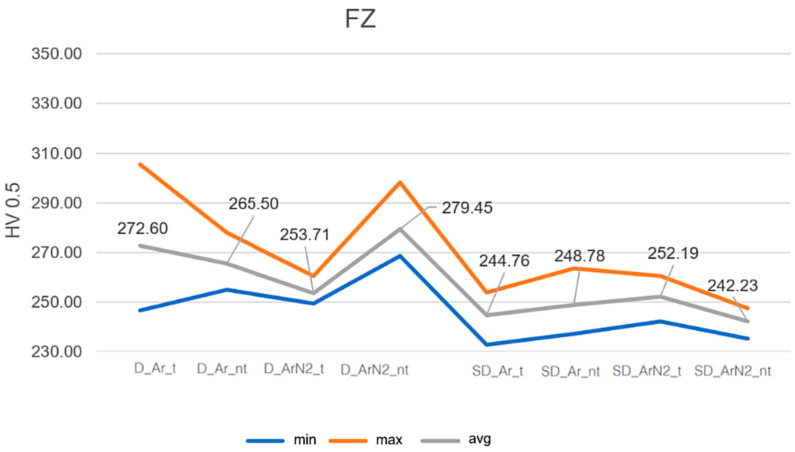
Microhardness in the FZ for D and SD samples.

**Figure 6:**
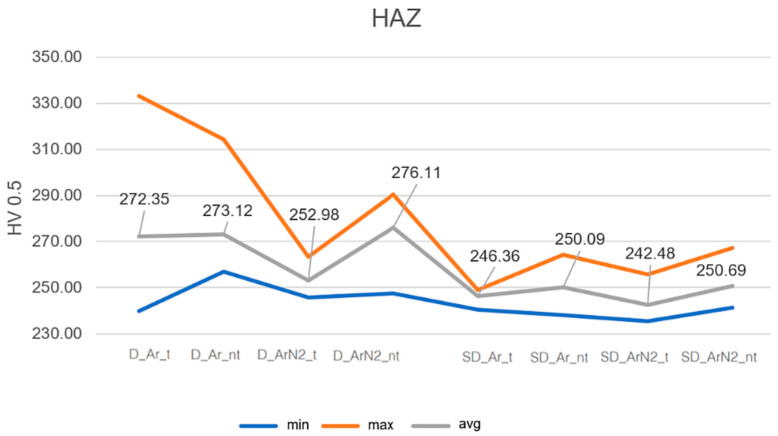
Microhardness in the HAZ for D and SD samples.

From such data, it has been possible to evaluate, for each sample, the average, maximum and minimum values of the HV0.5 to better show the variability of the measured values. Figure 5 and Figure 6 show the results, referring to the FZ and HAZ, respectively.

Results indicate that the microhardness in the FZ and HAZ is higher in the case of duplex stainless steels, as expected by the microstructural analysis. In the case of samples welded using the Ar2%N_2_ mixture the effect of the heat treatment is also evident, which induces a general decrease in the microhardness, probably correlated also to grain growth (the same effect can be seen in the Supplementary Materials, for the base metal).

In the case of super duplex samples, the microhardness differences induced by gas or heat treatment are less evident, and they can be considered practically constant within the experimental error.

### 3.3. Tensile Testing

**Figure 7:**
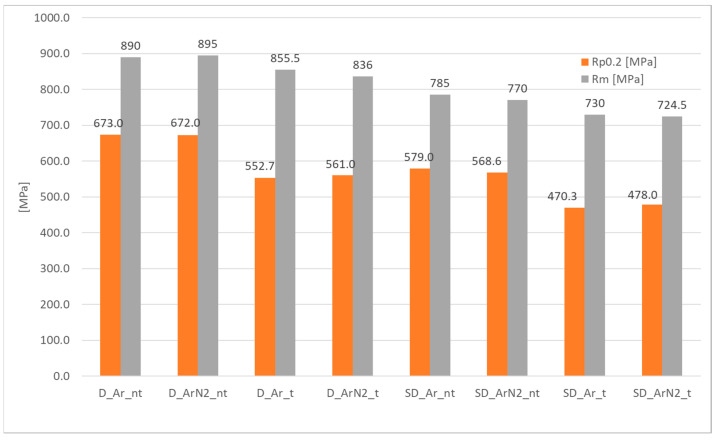
Average tensile (Rm) and yield (R_P0.2_) strength of the duplex (D_) and super duplex (SD_) samples.

Tensile test results are detailed in the Supplementary Materials, from which the chart of Figure 7 has been obtained, addressing the average yield strength (R_P0.2_) and tensile strength (R_m_) of the investigated samples. Only the average values are shown for clarity’s sake, but more detailed results on the repeated tests can be found in the Supplementary Materials regarding tensile testing.

Results confirm that duplex stainless steels present the higher yield and tensile strength for identical welding conditions. In the case of heat-treated samples (_t), in all cases, an average reduction of 5–7% of the tensile strength and of 17–20% of the yield strength is encountered. The gas mixture used has no practical effects on such quantities, both in the treated and untreated conditions. Hence, this reduction can likely be ascribed to the stress relief induced by the heat treatment, and possible defect healing, despite the short time of application.

Comparing the tensile tests results with literature data [30] and the data of the base metal, in the case of duplex steels, the welded samples present higher tensile and yield strength. In the case of super duplex stainless steels, similar values are achieved for the untreated samples, while slightly lower tensile and yield strength have been measured after heat treatment. Such results indicate that the parameters used for welding the duplex steels and the post-welding heat treatment do not significantly affect tensile and yield strength, while in the case of super duplex stainless steels, values approximatively 200 MPa lower are encountered.

**Figure 8:**
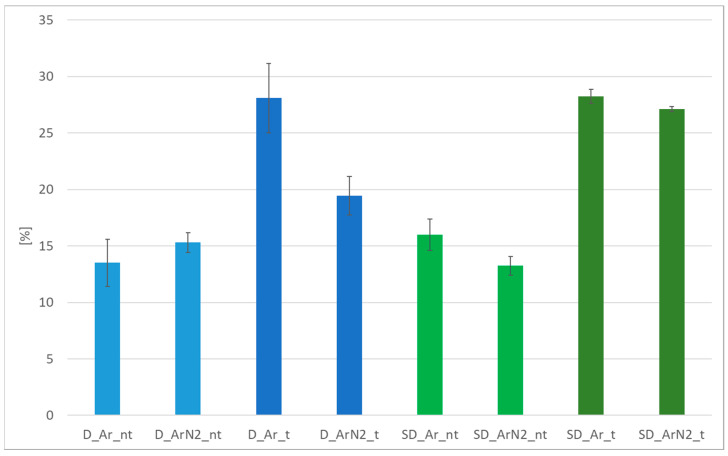
Average elongation at break (in percentage) of the duplex (D_) and super duplex (SD_) samples.

The average values of the elongation at break of each sample’s series are shown in Figure 8.

Results indicate that the heat treatment results particularly effective in increasing the elongation at break, which for all samples is increased by 45–50%, except for the case of the duplex samples welded in argon/nitrogen mixtures, for which the increase is 20%. The latter result is in contrast with the findings by Hosseini et al. [31], which reported an increase in the elongation at break in the case of argon/nitrogen mixtures. This effect is encountered in the preset study only in the case of untreated duplex stainless steel, while it does not apply to the other welding conditions investigated. Untreated samples present elongation at break significantly lower than the literature results, thus indicating the usefulness of the post-welding heat treatments to recover the plastic deformation capability of duplex and super duplex steels.

In a recent review by Mohammed et al. [36], the effects of heat input on microstructure and mechanical properties of duplex stainless steels are presented, concluding that a low heat input in the welding is responsible for a rapid cooling rate and, therefore, the depletion of austenite in the FZ and HAZ. The value of 0.5 kJ/mm, similar to the one used in the present study, is considered a low heat input [37]. In this case, such welding conditions can generate precipitation of the intermetallic, like Cr_2_N, because the high cooling rate makes it difficult for austenite to precipitate, and thus generates a greater amount of delta ferrite in the FZ and HAZ. Delta ferrite is more susceptible to precipitation as secondary phases of chromium nitrides (CrN_2_) and carbides, which are expected to adversely affect corrosion resistance as well [38,39]. In addition to CrN_2_, in these steels, depending on the heat treatment or welding process performed, other undesired intermetallic phases may form, which are detrimental to the mechanical properties and corrosion resistance [36,40].

Hence, such detrimental phases, which are known to form during welding [41], are responsible for the decrease in the elongation at break, creating preferential paths for cracks propagation. Literature results on the heat treatment of duplex stainless steel castings at temperatures similar to the ones present in the HAZ show that the sigma phase precipitates simultaneously with the secondary austenite. The interfaces between austenite or secondary austenite and the sigma phase are the locations where cracks, generated from the void aggregation, tend to propagate and to promote the sigma phase breaking off [40].

To better understand the positive effect of the post-welding heat treatment, it must be considered that precipitation of the sigma phase and other unwanted phases takes place as a function of the temperature and of the cooling rate, with secondary austenite, the sigma phase (σ) and the chi phase (χ) precipitation occurring during cooling from 950 to 600 °C, while the precipitation of the π, ε and α’ phases occurs between 300 and 600 °C [42]. Hence, post-welding heat treatments must be able to solubilize the unwanted phases and to avoid their re-formation by rapidly cooling the material, minimizing time spent in the critical 600–1050 °C range. However, during such treatment, the austenite and ferrite content are also affected, requiring a tradeoff between the formation of a more balanced phase balance and the removal of the sigma phase. Moreover, the heat treatment could be responsible also for the partial removal of residual stresses generated during welding or defect healing induced by the high temperatures. This is also evident in the reported microhardness values, which measure the local capability of the material to withstand plastic deformation, and which show a generally lower microhardness of heat-treated samples.

As indirectly shown in Figure 8, the heat treatments performed are effective in increasing the elongation at break, and the results of Figure 4 indicate that the treatment is successful in reducing the imbalance between ferrite and austenite. However, the success in the removal of the unwanted phases can be further confirmed by corrosion resistance testing.

### 3.4. Corrosion Tests

**Figure 9:**
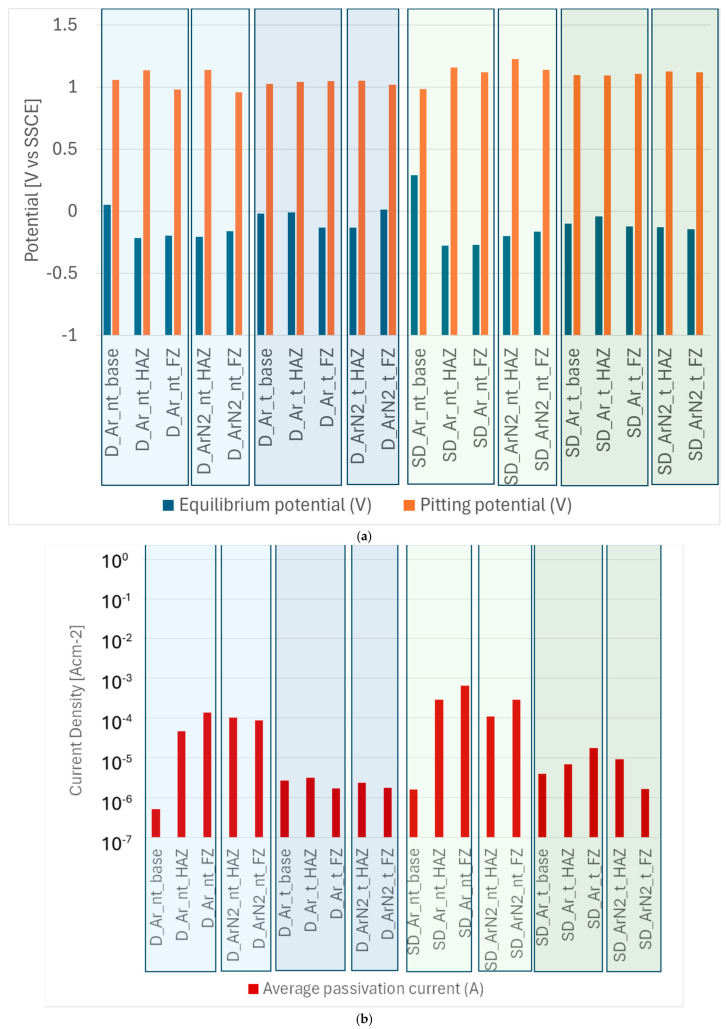
Corrosion tests results in terms of (**a**) equilibrium potential and pitting potential and (**b**) average passivation curve.

Polarization curves obtained for each sample were analyzed following the ASTM G5 standard to obtain the equilibrium potential (i.e., the potential at which the sample switches from cathodic to anodic behavior), the pitting potential (i.e., the potential from which a sudden increase in the anodic current is observed, an event associated, in this chloride-rich environment, with the formation of a pit) and the average passivation current (i.e., the average anodic current density obtained in the potential range from the equilibrium potential to the pitting potential). The results obtained are summarized in Figure 9.

Figure 9a highlights the high equilibrium potential of the base material, for both duplex (D_) and super duplex (SD_) samples. Noticeably, the post-welding heat treatment (_t) is responsible for a slight decrease in the equilibrium potentials in the base material. The most interesting result regarding equilibrium potentials is that, for the same alloy and heat treatment, the fused zone (FZ) and the heat-affected zone (HAZ) always show minimal differences, a result that allows us to exclude galvanic corrosion phenomena between the weld bead and the HAZ deriving from the use of filler material. Considering the pitting potential shown in Figure 9a, no major differences emerge, as all the tested samples maintain extremely high values of this parameter (very close and often higher than 1 V vs. SSCE), denoting a very high resistance to the pitting phenomenon in a marine environment. The presence of secondary phases (e.g., sigma phase) in duplex steels has a strong impact on the pitting potential of the alloy and exposes the material to the creation of galvanic microcells that trigger pitting corrosion in environments such as seawater [43,44]. The significant presence of sigma phase can, for example, cause the pitting potential to drop to values lower than 700 mV vs. SSCE [43,45]. The extremely high values of pitting potential found in all the conditions examined allow us to exclude the triggering of pitting phenomena in seawater at room temperature, given that the potential of the cathodic oxygen reduction reaction at neutral pH does not exceed 0.8 V vs. SSCE, thus remaining far lower than all the pitting potentials measured. These extremely high values of pitting potential, in agreement with [43], allow us to exclude the presence of secondary phases (for example, sigma phase) in quantities and distributions so significant as to trigger galvanic microcells and pitting. To better investigate the influence of welding and heat treatment processes on corrosion resistance, it is more useful to observe the average passivation current values.

Passivation current results plotted in Figure 9b show that the lower values, and hence the best passivation phenomena and the lower corrosion rate, are found in the case of base material, with minimum values often occurring in the FZ. It should be recalled that, according to the studies of Tavares et al. [28], the percentage of nickel in the filler material should be 2–4% higher than in the base material; this requirement was respected for either of the two materials investigated here, even if in the lower range, and this may have negatively affected the corrosion properties of the welded samples in the FZ.

Concerning the effects of the welding gas, no significant positive contribution by the addition of nitrogen is detectable in the case of duplex stainless steels, as it slightly improves the performance of the FZ, while it worsens that of the HAZ. Conversely, in the case of super duplex stainless steels, the beneficial effect is very evident since the values of average passivation current are halved. This result is in agreement with the reduction in ferrite percentage observed by the microstructural analysis: in the presence of nitrogen, a lower percentage of ferrite is encountered in both the FZ and HAZ, with the FZ having almost the same ferrite content of the base metal.

The performed heat treatment resulted particularly beneficial to increase the corrosion resistance of the samples. Both classes of materials improved their performance by one order of magnitude compared to their untreated counterparts. This is a very interesting result, and, in part, contradicts the findings of previous work on post-weld treatment carried out at 1000 °C for less than 10 min, which successfully rebalanced the two phases but did not improve corrosion properties in the case of TIG welding [46]. However, in the study by Cho et al. [47], the poor performances of heat-treated TIG welds were ascribed to the formation of oxides, indicating possible problems in the shielding gases or the welding procedure, not occurred in our study. Phase rebalancing was also observed in another recent study [48], in which a heat treatment of 1 h at 1050 °C increased the austenite percentage to 48.4%. The elimination of secondary precipitated phases generated a more uniform structure, which provided better corrosion resistance, in agreement with the results of the present study.

The comparison between the two materials investigated is also interesting: welded duplex stainless steels show performance comparable to, if not slightly better than, super duplex ones. As a matter of fact, the corrosion performance of the HAZ and the FZ deteriorated significantly compared to what occurred with duplex samples.

## 4. Conclusions

The post-welding heat treatment at 1120 °C for 1 h in an argon atmosphere at 6 bars produced two beneficial effects: firstly, it significantly improved corrosion resistance, restoring it almost to the original level of the unwelded base material in both alloys investigated; secondly, it nearly doubled the elongation at break of both duplex and super duplex stainless steels.

In the case of marine or offshore applications, super duplex steel is often chosen over duplex steel for welded constructions due to its presumed higher corrosion resistance. However, if welding is not performed correctly, it can result in products with properties well below those found in the literature, thus negating the use of higher quality and more expensive materials.

The present study allows us to have a deeper insight into the effect of different welding parameters such as heat input, gas shielding and post-welding heat treatment on mechanical properties and corrosion behavior of UNS S 31803 (duplex) and UNS S 32760 (super duplex) stainless steel. Based on the experimental results, the following conclusions can be drawn:

- To produce all the samples, steel plates in DSS and SDSS with Y-shaped edges were chosen so that the welded joint could be made in a single pass. During the execution of the joints, it was found that by using Ar + 2%N_2_ shielding gas, the welding current can be reduced by 10% compared to when pure Ar is used. However, for all samples produced, the heat input transferred to the material has been set in order to stay within the range of 0.48–0.56 kJ/mm.
- Although the literature recommends using filler materials with nickel content 2–4% higher than the base materials for welding SDSS and DSS steels, in this study, we investigated the possibility of using rods made of the same alloy of the base material. The purpose of this approach is to produce test specimens with a homogeneous chemical composition which, especially in the case of PWHT, are intended to produce welded parts with high mechanical strength and superior corrosion resistance. This objective was achieved only by the duplex steel samples, as the super duplex steel samples showed lower mechanical properties and corrosion resistance than initially expected.
- The use of shielding gas containing 2% nitrogen compared to traditional argon led to different effects in the weld area between the two materials considered. In the case of DSS, with both shielding gases the ratio between the two phases is approximately 32% ferrite/68% austenite, so no significant benefits can be identified; this aspect is confirmed also from the tensile test data, in which as weld conditions are practically identical. After solution heat treatment, the behavior differs, and the specimen welded with the Ar + 2%N_2_ mixture shows higher content of ferrite (58% vs. 44%) with respect to the one welded in pure Ar. This difference in phases distribution plays a role mainly on the total elongation, which is significantly higher for the specimen welded in pure Ar; in this case, the elongation reaches 28%, which is above the minimum value required for the raw material UNS S 31803. Instead, in the case of SDSS, it is possible to detect in the specimens in “as weld” condition a significant difference in phase balancing, depending on the shielding gas used. In detail, the use of the Ar + 2%N_2_ mixture helps in maintaining a better ratio ferrite/austenite (59–41%), while the specimens welded in pure Ar shielding appear to be completely out of balance from a microstructural perspective (22% austenite/78% ferrite). Despite this result, it should be noted that the elongation values specimens in the “as-welded” state are still well below the typical values for this steel and, above all, are almost a half of those specified in the reference standard. After solution heat treatment, the phase balance becomes, in both cases, perfectly balanced, with comparable values and the elongation rises to 27/28%. However, the mechanical characteristics measured with longitudinal tensile tests are approximately 15% lower than those required by the relevant standard.
- The effect of shielding gas can also be considered in relation to corrosion resistance near the welded joint. Corrosion tests evidence that, for DSS steels, no positive contributions are found in the use of Ar + N_2_ gas; the performance of the welded area improves slightly, while the ZTA is the area with the worst performance. On the other hand, in the case of SDSS, the beneficial effect of Ar + N_2_ shielding gas is more pronounced, as the average passivation current values are halved. This result can be explained by the decrease in the amount of ferrite observed in SDSS, as highlighted in the previous paragraph.
- In terms of corrosion resistance, the comparison between the two materials under investigation underlined an unexpected behavior: welded DSS using the parameters investigated in the present study has comparable, if not slightly superior, performance compared to SDSS. In SDSS, the corrosion performance of the HAZ and weld area is significantly lower compared to DSS. On the other side, according to the studies by Tavares et al. [28], the percentage of nickel in the filler material should be 2–4% higher than in the base material; this was respected in the present study for both materials, but with a limited excess of Ni (2.3% more for DSS and 3.1% more for SDSS compared to the base materials), and this may have had a negative effect on the corrosion properties of the SDSS-welded samples.
- Considering the effect of the solution heat treatment on the corrosion performance of the samples, both materials improved their performance by an order of magnitude compared to their untreated counterparts. This is a very interesting result as it is not reflected in some of the mentioned literature studies, which report that the post-welding treatment carried out at 1000 °C for less than 10 min, while rebalancing the two phases, did not improve the corrosion resistance properties in the case of TIG welding.

Both the mechanical and corrosion resistance performances of welded super duplex stainless steels were found to be inferior to those of duplex ones. In the case of the mechanical performance of the welded specimens, this may be attributed to the precipitation of deleterious secondary phases, which cannot be detected with the experimental techniques employed in this study and will be subject of further investigations.

The results obtained provide valuable insights for selecting the appropriate welding procedure depending on the final application. Concerning post-welding solution annealing treatments, their beneficial effect was demonstrated, but they could have limited applicability in the case of complex-shape parts, requiring complying with strict dimensional and geometric tolerances, which could be altered by the treatment applied in this study.

## Figures and Tables

**Table 1:** Chemical composition of the samples (Fe = bal).

	C %	Mn %	P %	S %	Si %	Cr %	Ni %	Mo %	N %	Cu %	W %
**Duplex UNS S31803**	0.019	1.33	0.027	0.001	0.40	22.5	5.6	3.2	0.18	-	-
**Super duplex UNS S32760**	0.015	0.67	0.028	0.001	0.32	25.19	6.99	3.50	0.272	0.55	0.53

**Table 2:** Chemical composition of the filler materials (Fe = bal).

	C %	Si %	Mn %	P %	S %	Cr %	Ni %	Mo %	N %
**S31803–ER 2209**	0.009	0.549	1.5	0.022	0.001	22.96	8.79	3.1	0.16
**S32760-ER 2594**	0.014	0.41	0.6	0.013	0.001	25.17	9.33	4.02	0.26

**Table 3:** Welding parameters.

Materials and Gases	Current [A]	Voltage [V]	Welding Speed [mm/s]	Arc Linear Energy Density [kJ/mm]
Duplex Ar	100	9.5	2	0.48
Duplex Ar + N_2_	90	9	1.5	0.54
Super Duplex Ar	110	10.5	2	0.58
Super Duplex Ar + N_2_	90	9	1.5	0.54

## Data Availability

The original contributions presented in this study are included in the article/Supplementary Materials. Further inquiries can be directed to the corresponding author.

## Supplementary Materials

The following supporting information can be downloaded at: https://www.mdpi.com/article/10.3390/ma18214818/s1, File S1: microhardness profiles and micrographs of the welded regions of samples for samples D_Ar_nt, D_ArN2_nt, D_Ar_t, D_Ar_nt, SD_Ar_nt, SD_ArN2_nt, SD_Ar_t and SD_ArN2_nt; File S2: tensile tests results for samples D_Ar_nt, D_ArN2_nt, D_Ar_t, D_Ar_nt, SD_Ar_nt, SD_ArN2_nt, SD_Ar_t and SD_ArN2_nt.
