# Supplementary material for: Effect of Shielding Gas and Post-Welding Heat Treatment on the Mechanical and Corrosion Performances of Duplex and Super Duplex Stainless Steels’ Low Heat-Input Welded Joints
Source: Materials (Basel). 2025 Oct 22;18(21):4818. doi: 10.3390/ma18214818 (PMC12609741; doi:10.3390/ma18214818)
Supplement: Supplementary file 1 [file materials-18-04818-s001.zip › materials-3893589-supplementary File S2.pdf]

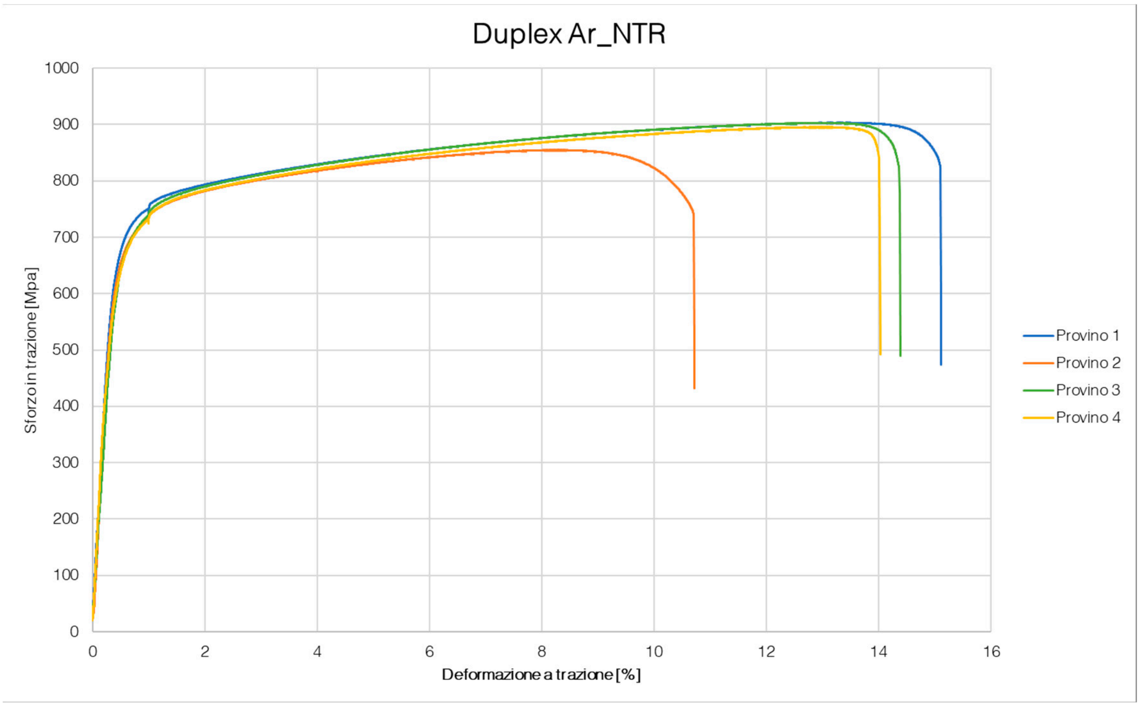

***Figure S1**: stress–strain curves of 4 specimen D_Ar_nt*

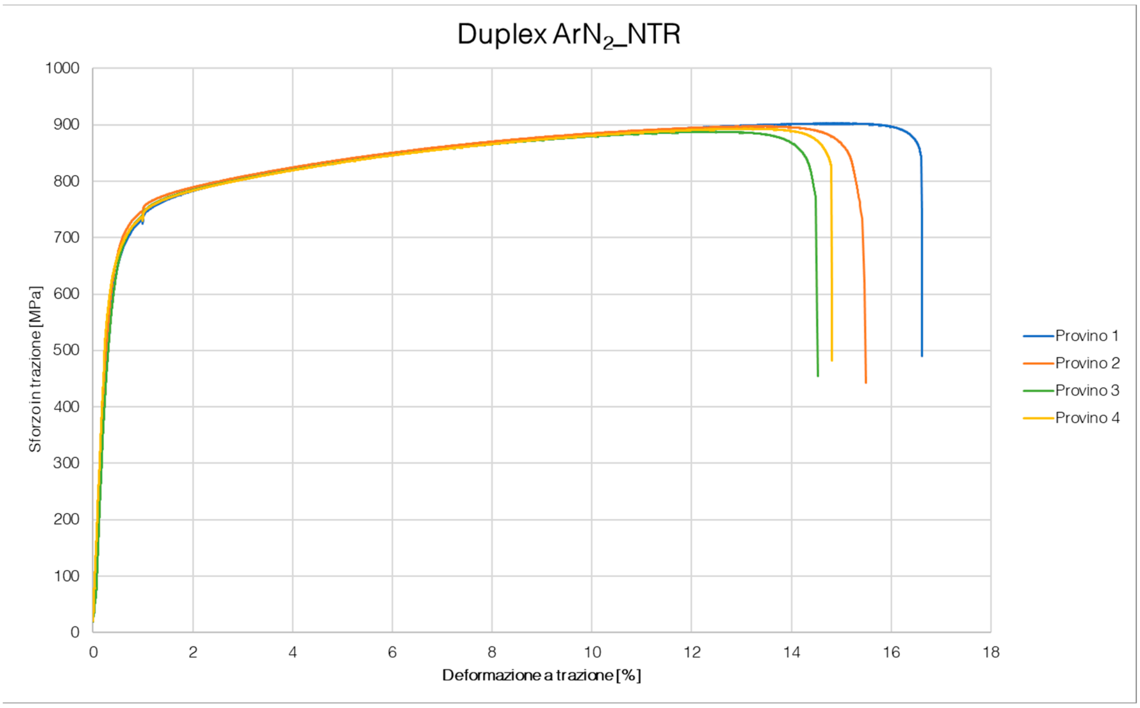

***Figure S2**: stress–strain curves of 4 specimen D_ArN2_nt*

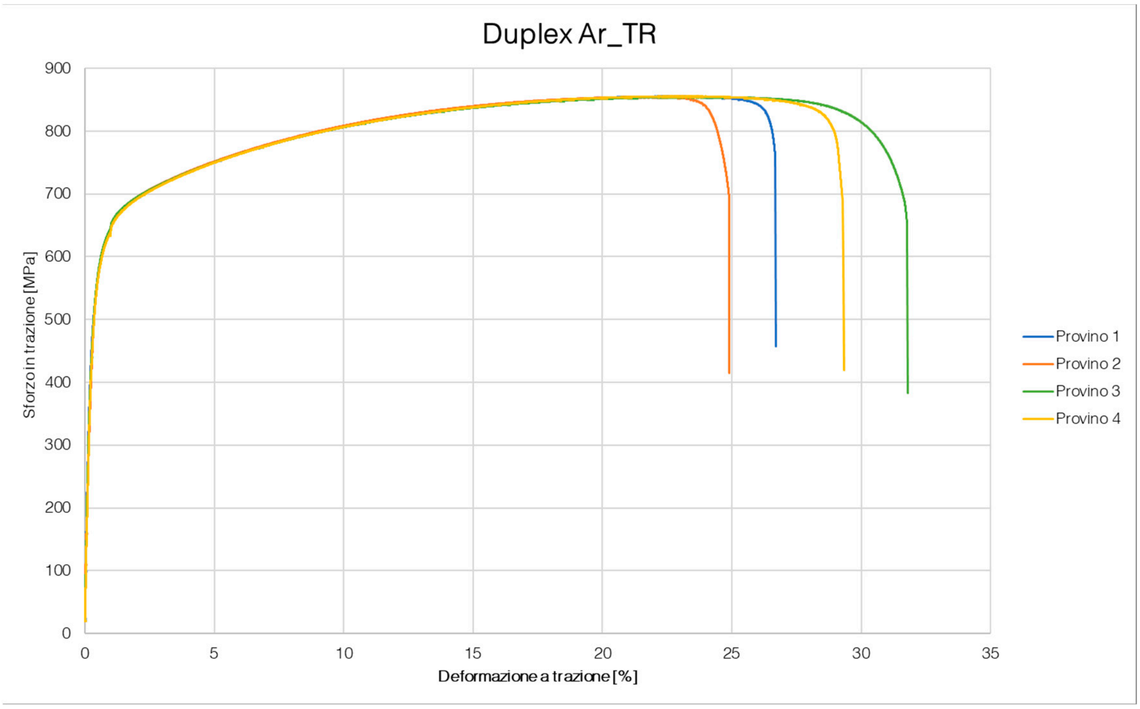

***Figure S3**: stress–strain curves of 4 specimen D_Ar_t*

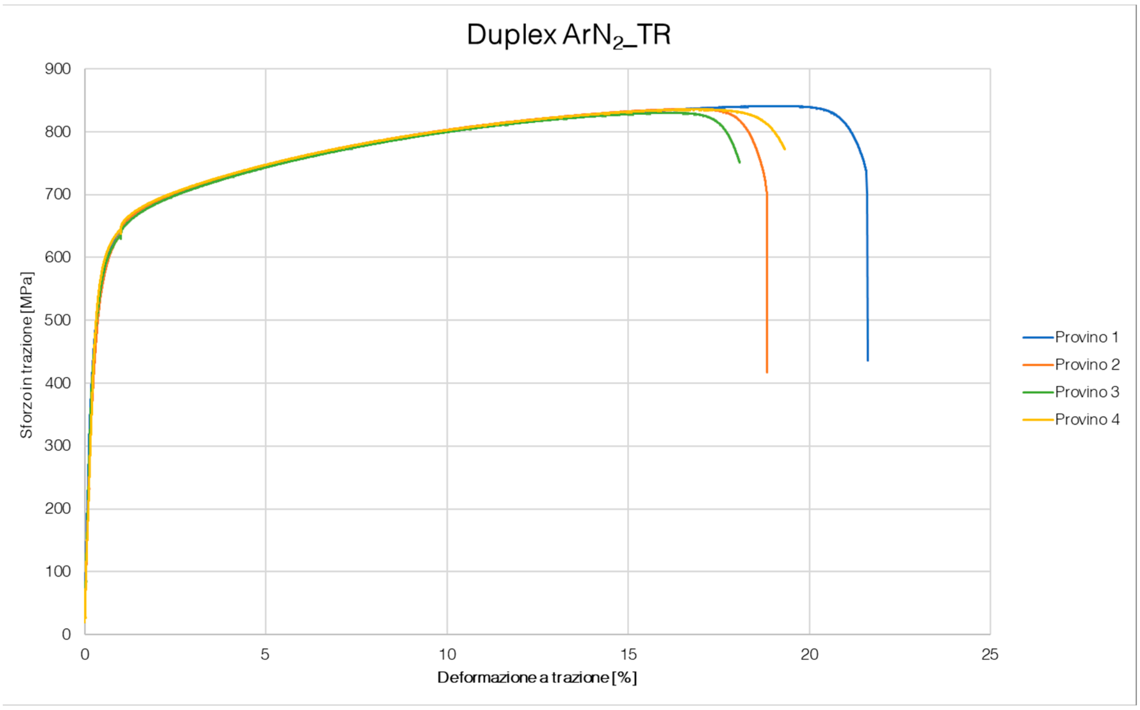

***Figure S4**: stress–strain curves of 4 specimen D_Ar_nt*

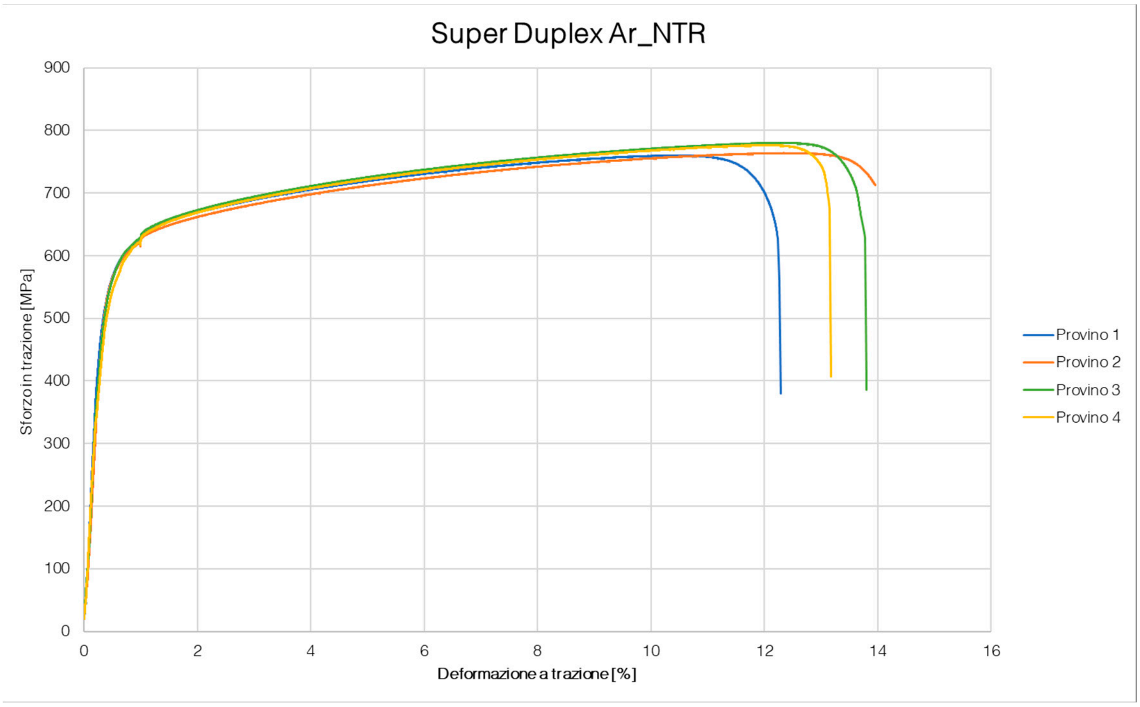

Figure S5*: stress–strain curves of 4 specimen SD_Ar_nt*

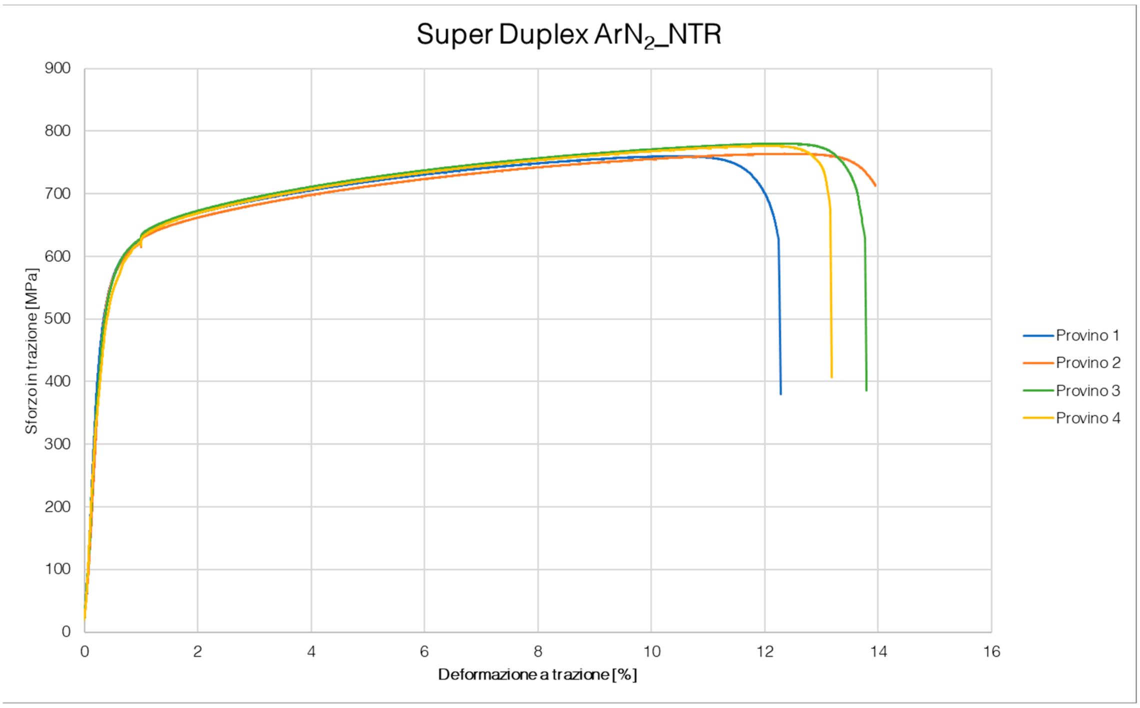

Figure S6*: stress–strain curves of 4 specimen SD_ArN2_nt*

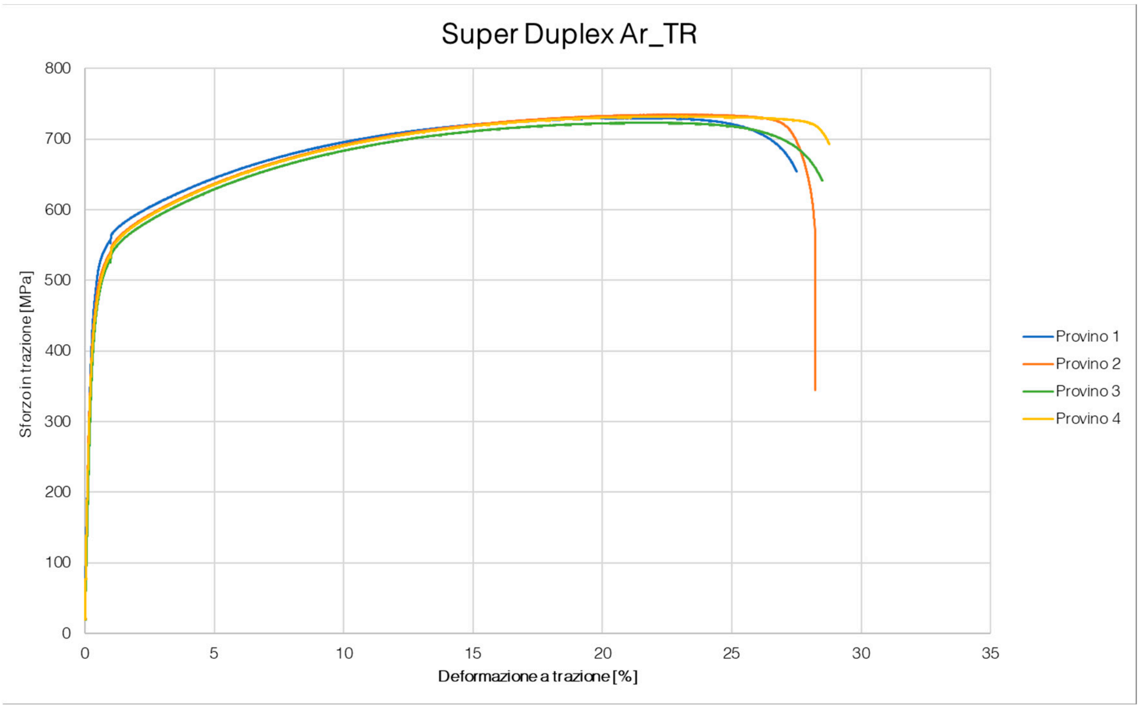

***Figure S7**: stress–strain curves of 4 specimen SD_Ar_t*

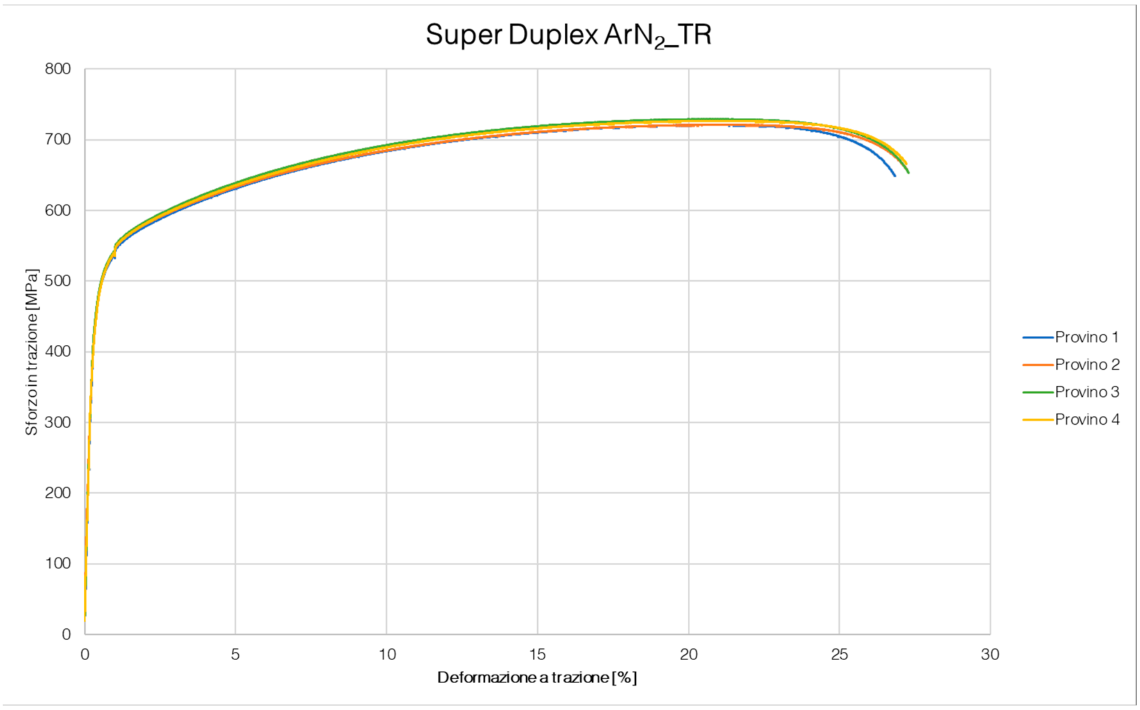

***Figure S8**: stress–strain curves of 4 specimen SD_ArN2_nt*
