# Supplementary figures and images for: Effect of Shielding Gas and Post-Welding Heat Treatment on the Mechanical and Corrosion Performances of Duplex and Super Duplex Stainless Steels’ Low Heat-Input Welded Joints
Source: Materials (Basel). 2025 Oct 22;18(21):4818. doi: 10.3390/ma18214818 (PMC12609741; doi:10.3390/ma18214818)

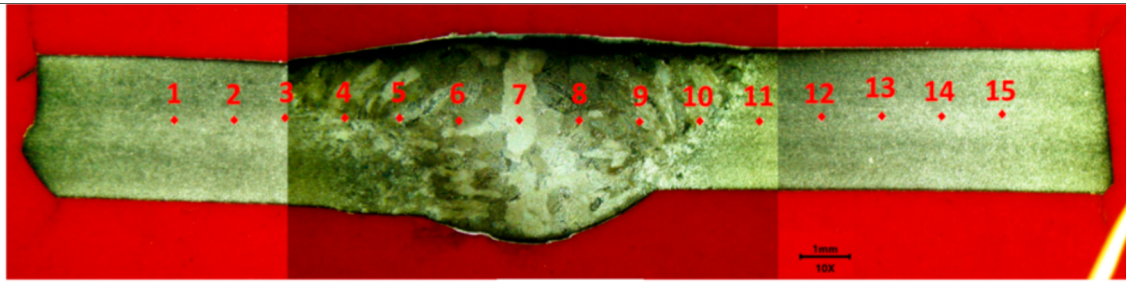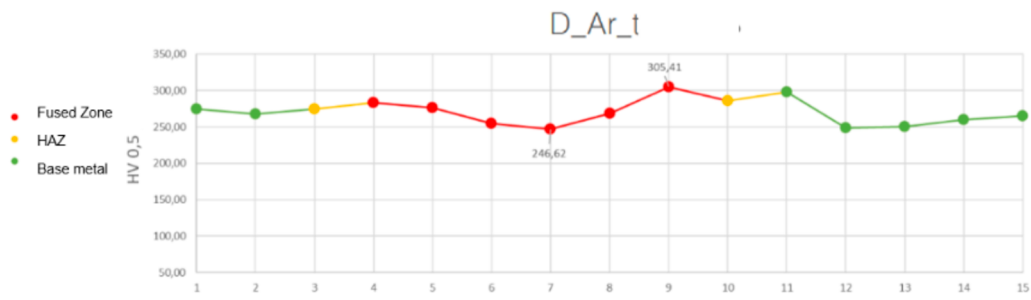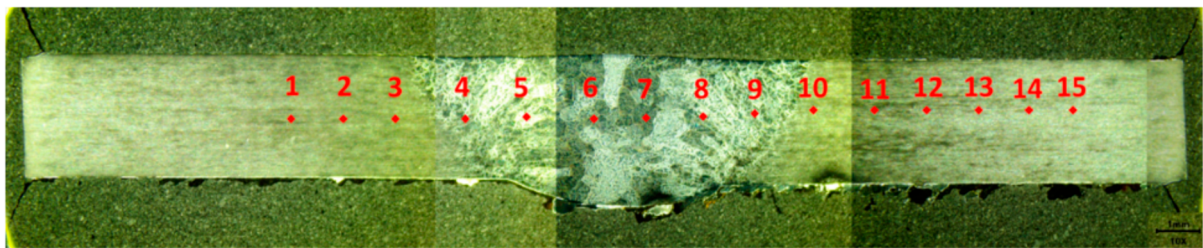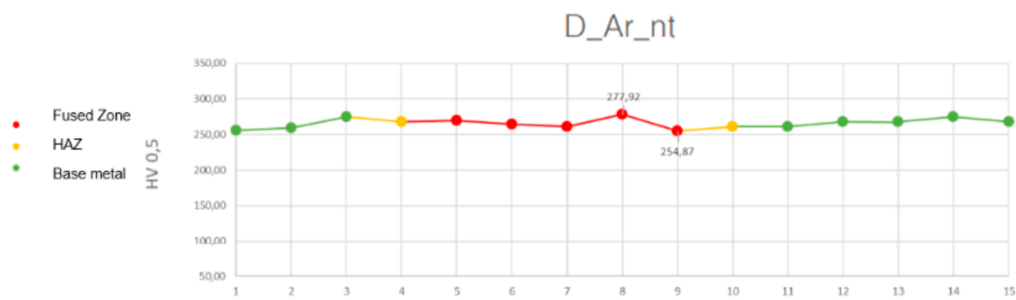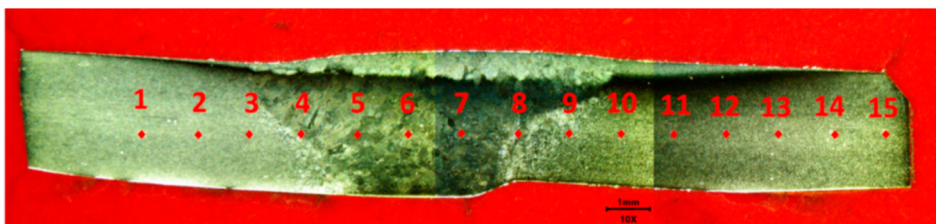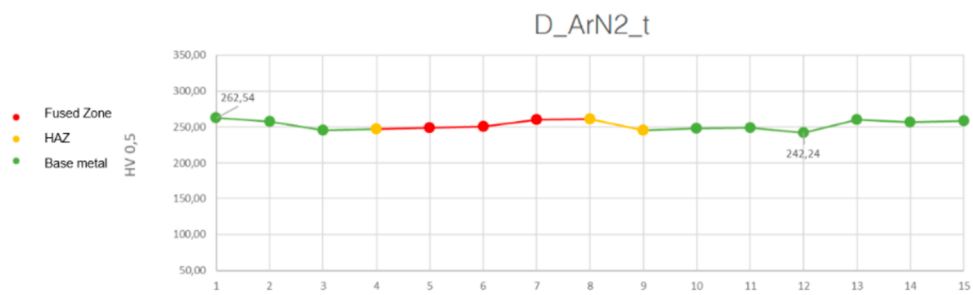

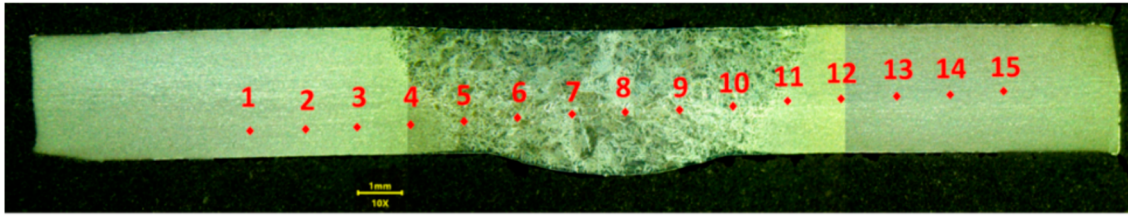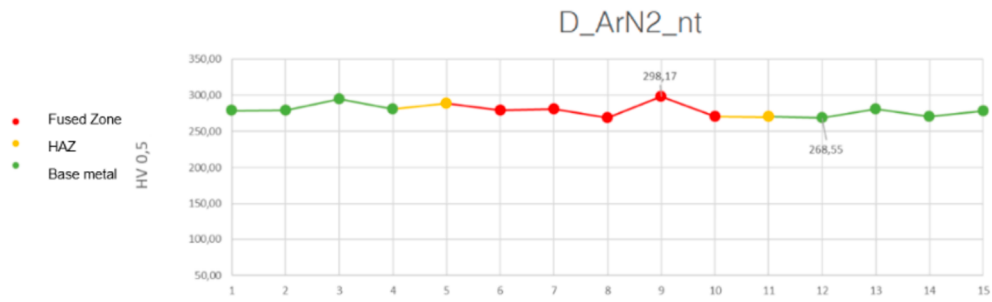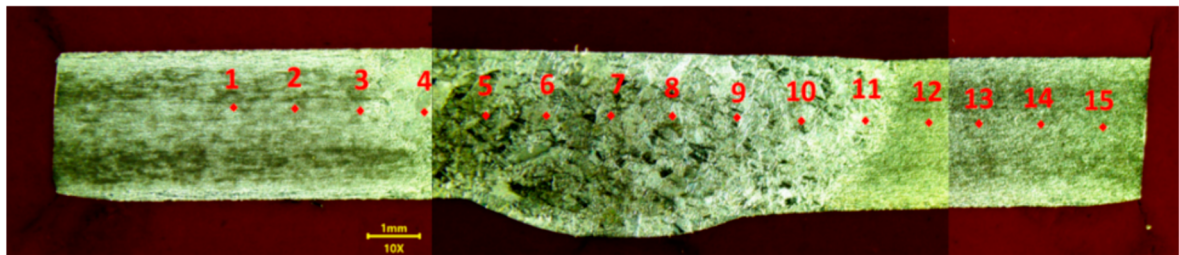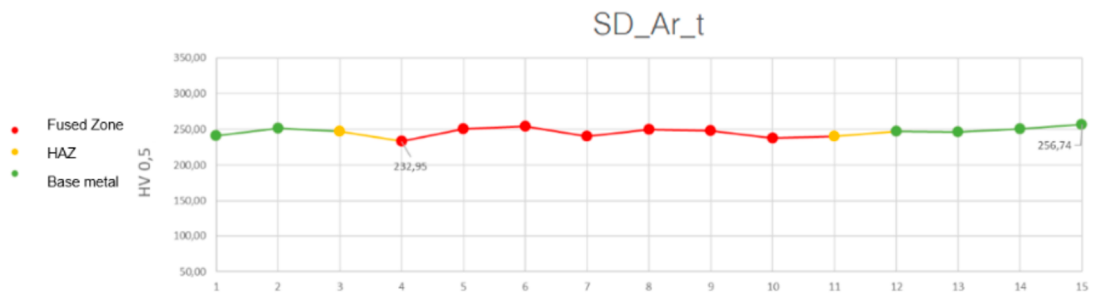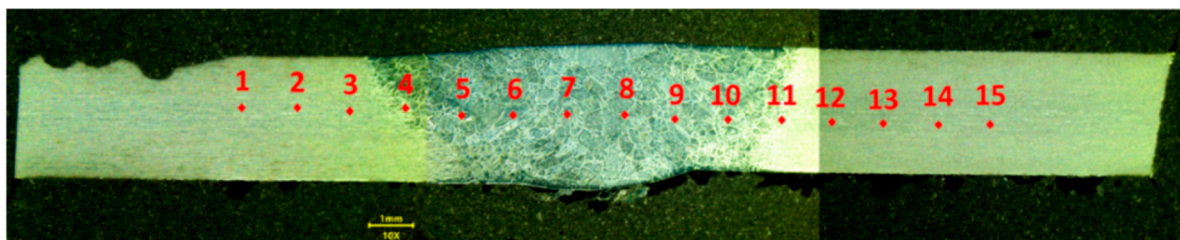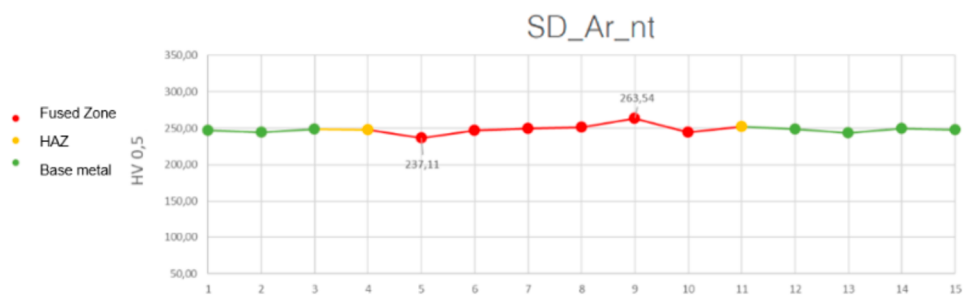

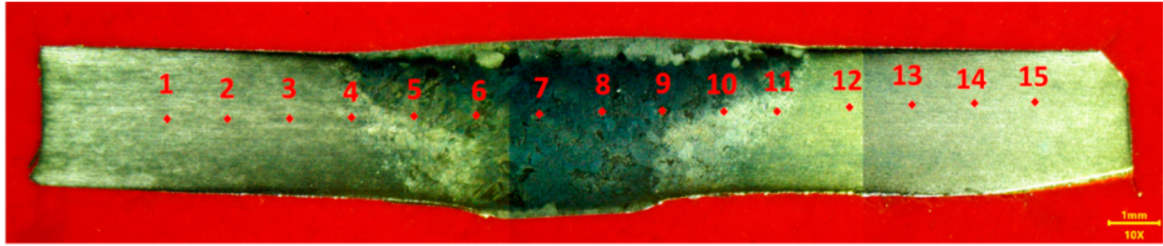

SD\_ArN2\_t

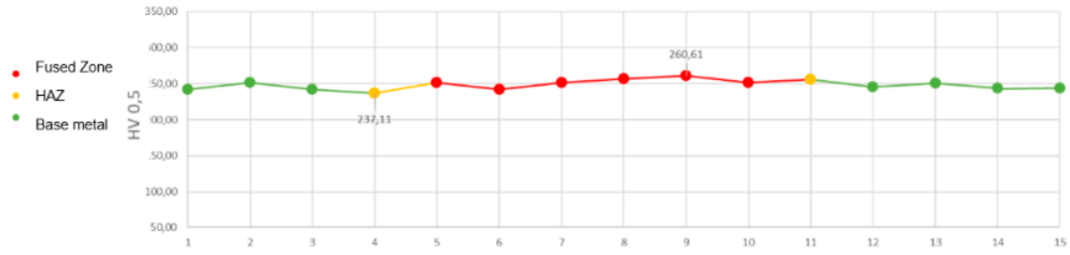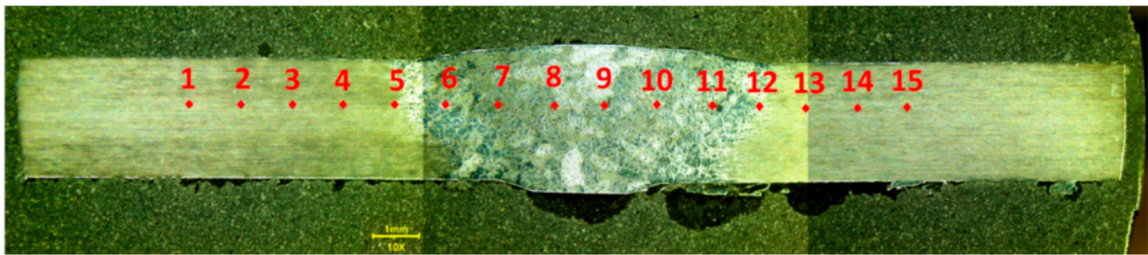

SD\_ArN2\_nt

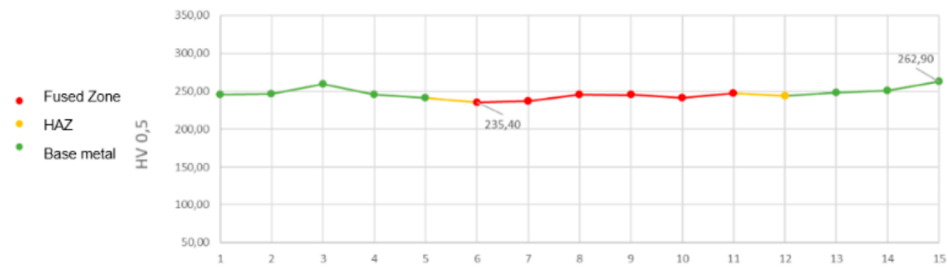

Supplement: Supplementary file 1 [file materials-18-04818-s001.zip › materials-3893589-supplementary File S1.pdf]
